# Somatosensory Plasticity in Pediatric Cerebral Palsy following Constraint-Induced Movement Therapy

**DOI:** 10.1155/2018/1891978

**Published:** 2018-11-08

**Authors:** Pawel J. Matusz, Alexandra P. Key, Shirley Gogliotti, Jennifer Pearson, Megan L. Auld, Micah M. Murray, Nathalie L. Maitre

**Affiliations:** ^1^The LINE (Laboratory for Investigative Neurophysiology), Radiology Department and Neuropsychology Service, University Hospital Center and University of Lausanne, 1011 Lausanne, Switzerland; ^2^Information Systems Institute at the University of Applied Sciences Western Switzerland (HES-SO Valais), 3960 Sierre, Switzerland; ^3^Department of Hearing and Speech Sciences & Vanderbilt Kennedy Center, Vanderbilt University Medical Center, Nashville, TN, USA; ^4^Department of Pediatric Rehabilitation, Vanderbilt University Medical Center, Nashville, TN, USA; ^5^School of Health and Rehabilitation Sciences, The University of Queensland, Brisbane, QLD, Australia; ^6^EEG Brain Mapping Core, Center for Biomedical Imaging (CIBM), University Hospital Center and University of Lausanne, 1011 Lausanne, Switzerland; ^7^Department of Ophthalmology, University of Lausanne, Fondation Asile des Aveugles, Lausanne, Switzerland; ^8^Department of Pediatrics and Center for Perinatal Research, Nationwide Children's Hospital, Columbus, Ohio, USA

## Abstract

Cerebral palsy (CP) is predominantly a disorder of movement, with evidence of sensory-motor dysfunction. CIMT^1^ is a widely used treatment for hemiplegic CP. However, effects of CIMT on somatosensory processing remain unclear. To examine potential CIMT-induced changes in cortical tactile processing, we designed a prospective study, during which 10 children with hemiplegic CP (5 to 8 years old) underwent an intensive one-week-long nonremovable hard-constraint CIMT. Before and directly after the treatment, we recorded their cortical event-related potential (ERP) responses to calibrated light touch (versus a control stimulus) at the more and less affected hand. To provide insights into the core neurophysiological deficits in light touch processing in CP as well as into the plasticity of this function following CIMT, we analyzed the ERPs within an electrical neuroimaging framework. After CIMT, brain areas governing the more affected hand responded to touch in configurations similar to those activated by the hemisphere controlling the less affected hand before CIMT. This was in contrast to the affected hand where configurations resembled those of the more affected hand before CIMT. Furthermore, dysfunctional patterns of brain activity, identified using hierarchical ERP cluster analyses, appeared reduced after CIMT in proportion with changes in sensory-motor measures (grip or pinch movements). These novel results suggest recovery of functional sensory activation as one possible mechanism underlying the effectiveness of intensive constraint-based therapy on motor functions in the more affected upper extremity in CP. However, maladaptive effects on the less affected constrained extremity may also have occurred. Our findings also highlight the use of electrical neuroimaging as feasible methodology to measure changes in tactile function after treatment even in young children, as it does not require active participation.

## 1. Introduction

Cerebral palsy (CP) is a disorder of movement originating from perinatal insults to the developing brain, with an incidence of 2–3 children per 1000 in the developed world. While CP is predominantly characterized by neuro-motor abnormalities, recent research has demonstrated the essential role of somatosensory system dysfunction in impaired movement generation. Abnormal processing of somatosensory stimuli is prevalent in children with CP and contributes to poor cortical feedback during probabilistic learning of movement, by providing imprecise or incorrect inferential data [1–3] In the case of hemiplegic or markedly asymmetric forms of CP, the less-affected hand can exhibit significantly different processing of somatosensory stimuli compared to the one controlled by a more lesioned cortical hemisphere. Greater motor and strength differences between more and less-affected hands are often correlated with worse somatosensory function [3–5].

To date, the effectiveness of rehabilitation strategies in recovering motor function in impaired extremities has been established primarily through functional assessments of movement [6]. Recovery of sensory function is difficult to measure consistently using behavioral responses to stimuli in children with CP under 10 years of age [4]. Few studies have attempted to elucidate the neural mechanisms underlying sensory and sensory-motor changes following childhood rehabilitation, largely due to the challenges of studying young children with disabilities using neuroimaging. ERPs and their MEG counterparts (i.e., event-related fields, ERFs) have allowed scientists to examine cortical processing of sensory stimuli in children with CP. Recent studies have demonstrated the feasibility of using such tools to investigate neural changes following evidence-based treatments such as constraint-induced movement therapy or CIMT [7]. The high spatio-temporal resolution of MEG also allowed identification of wide-ranging oscillatory abnormalities within the somatosensory cortex of the affected hemisphere [3, 8, 9]. Specifically, attenuated oscillatory scalp and brain activity has been consistently reported in more versus less affected somatosensory cortices in hemiplegic CP. The extent of these impairments predicted motor performance across a wide range of functions, suggesting dysfunctional activity within the somatosensory system as one of the underlying core deficits. Our previous event-related potential (ERP) study complemented these findings by replicating the asymmetries in somatosensory activity, while also demonstrating the poststimulus timing, the clinical importance, and the behavioral relevance of brain responses to somatosensory stimuli [5]. An electrical neuroimaging approach to the analysis of ERPs can further leverage the spatio-temporal information in the ERP data to clarify if alterations in brain source configuration or strength of response of the same network contribute to motor function deficits and/or their recovery in pediatric CP. These mechanistic insights are afforded by robust, reference-independent, and global analyses of the electric field at the scalp (e.g., [10, 11]). Electrical neuroimaging analyses have been invaluable for identifying temporal and spatial mechanisms governing plasticity of sensory representations in both healthy (e.g., [12–14]) and developmentally atypical subjects [15, 16].

Building on the previous M/EEG findings, in the current study, we hypothesized that in CP, altered somatosensory processing is central to sensory-motor deficits and that ERPs can capture asymmetries in somatosensory function usually diagnosed only with behavioral tools [5]. In the present study, we aimed to test whether the analytic approach within the electrical neuroimaging framework [10] can elucidate mechanisms of plasticity of the somatosensory cortical network and their contribution to improvements in sensory-motor function in CP (i.e., in the more affected extremity). Because EEG-based testing of infants and young children is rapid and highly feasible, electrical neuroimaging may then constitute an appropriate methodology for evaluation of CP therapeutic strategies, such as CIMT. CIMT is effective in children, yet still requires extensive optimization [17]. Therefore, elucidation of somatosensory changes and their brain underpinnings following treatment can suggest mechanisms contributing to sensory-motor impairments and, thus, the design of new and more effective clinical interventions. In particular, when CIMT involves prolonged wear of a hard nonremovable cast over the less affected hand, the use of the more affected extremity may be promoted at the expense of decreased sensory input to the less affected one. Notably, it currently remains unclear whether this lack of sensory input alters somatosensory processing in the less affected extremity. If demonstrated in our study, such a loss may suggest the need for adjustments to the type and/or intensity of CIMT protocols. We therefore investigated whether alterations in somatosensory processing following intensive nonremovable hard-constraint CIMT in children with hemiplegic CP can be quantified using electrical neuroimaging framework analyses of the spatio-temporal characteristics of ERP responses to a calibrated light touch. We designed a prospective interventional study of young children undergoing a week-long CIMT intervention previously shown to improve upper limb function [7]. We compared changes in cortical processing of touch pre- and post-CIMT, as well as between more and less affected sides. In relation to the more affected hand, our predictions were the following. If intensive CIMT acts predominantly through increasing the gain-response function within a somatosensory network poorly responsive to (light) touch, its effects should be seen exclusively as stronger touch-induced responses, measured as GFP differences between pre- vs. post-CIMT ERPs. In contrast, if CIMT acts predominantly by changing a dysfunctional pattern of somatosensory activity, the timing and topography of ERPs recorded after the treatment should resemble those in the less affected hand prior to CIMT. Note that these possibilities are not mutually exclusive. Our differential predictions for the CIMT-induced effects in the less affected hand mirror those for the more affected hand: if CIMT has a negative effect on the relatively more healthy somatosensory system and acts by modulating the responsiveness of a somatosensory brain network processing touch, intensive CIMT will exclusively reduce the strength of ERPs in the less affected hand upon light touch. Alternatively, if CIMT acts by altering patterns of brain activity even when they are more functional, ERPs upon stimulation of the less affected hand would instead show pre- vs. post-CIMT differences in timing and topography.

## 2. Materials and Methods

### 2.1. Participants

Ten children with CP with a mean age of 6.5 years old (SD 1.1 years), 50% female, with four subjects born preterm (<37 weeks gestational age), were included in the present study (Table 1). Manual Abilities Classification score (MACS) range was 1–3. All assessments were performed immediately prior to placement of the constraint (preintervention) and 2 hours after its removal (postintervention). Inclusion criteria were the following: (1) medical diagnosis of CP with asymmetric deficit of tone, posture, and movement in one upper extremity as determined in the pediatric neurology clinic using standard care neurological exams, (2) age 5–8 years, (3) toilet training, and (4) a score ≥ 60 on the nonmotor domains of the 60-month Ages and Stages Questionnaire. Patients with uncontrolled seizure disorder or botulism toxin injection in upper extremities within 6 months of the start of the study were excluded. All parents provided a written informed consent, and all children assented to the study using Institutional Review Board-approved protocols and forms. All the procedures were carried out in accordance with the Declaration of Helsinki for experiments involving human participants.

### 2.2. Constraint

After preintervention assessments, a therapist covered the less affected extremity with a nonremovable rigid cast from the proximal humerus to the proximal phalanx of the fingers, maintaining the wrist in a neutral position and the hand open. After the 5-day group therapy intervention over the course of one week, the cast was removed, and parents received suggestions for continued use of the more affected extremity as well as a removable cast. Total cast-wearing time was 120 hours per child and included 22 hours of goal-directed activities and shaping of movements by occupational and physical therapists. Daily programs consisted of gross motor/bilateral activities focused on balance and proprioception, fine motor activities, and unilateral self-care activities. Children also participated in fine motor activities that involved a sensory feedback component such as temperature, texture, light and deep pressure, and vibration. These interventions totaled 10 hours over the course of the week.

We employed the less affected limb as a *within-subject* control in our study. For one, there were severe potential ethical concerns in using a healthy group of children as controls due to safety concerns regarding the effects of the constraint on normal tactile and motor function at a critically sensitive stage (e.g., [18, 19]). To foreshadow our findings, we found evidence for less functional patterns of activity in the less affected hand post-CIMT. As it cannot be assured that CIMT would not result in unwanted potentially long term functional effects, using the other limb in children with hemiplegic CP as a control was least ethically doubtful.

### 2.3. Sensory, Sensory-Motor, Range of Motion, and Strength Assessments

An expert in behaviorally based sensory assessments [4] designed the approach for this study, with the modification of using a screen separating the child's arm and hand from vision for all testing. Somatosensory registration was tested using the Semmes Weinstein monofilament (SWM; AliMed, Dedham MA) kit. The final score was the lowest monofilament value at which the child was able to correctly identify at least one touch (out of a possible three plus one sham control for each filament) on the distal phalanx of the index finger. Single-point localization was assessed on lateral, dorsal, and ventral surfaces of the same finger using the largest SWM and a sham touch. The final score was the average number of correct responses for each possibility, with a range 0–4 possible. Static two-point discrimination was tested using a Disk-criminator (AliMed, Dedham MA) on the distal pad of the index finger. Children were asked whether they felt one point or two points over the course of ten random applications covering the range of 1 to 20 mm; the final score was the lowest distance in mm at which two distinct points were reported. Stereognosis was tested with children placing both hands through the holes in a screen with elbows resting on a table and being asked to look at a board with 9 object pairs that were moderately similar in shape and size but different in texture, weight, or somatosensory details (e.g., wooden button with holes/metal coin, plastic spoon/fork). Objects were pointed at and named by the examiner and repeated by the child. The examiner then presented one of each pair to the child behind the screen and asked the child to name the object for a total of 9 presentations. The final score was the number of correctly identified pairs from 0 to 9. Because grip and pinch are sensory-motor tasks [18, 20], they were also measured, using a standard adjustable-handle Jamar dynamometer and a B&L pinch gauge [21]. Scores were reported as the maximum pressure exerted of three attempts with the child being asked to pinch or grip as hard as they felt they could. However, the grip referred to here is a measure of strength and not exclusively a sensory task. Nonetheless, the sensory system likely influences the production of maximal voluntary contractions (e.g., [22]). Extending the previously used model of CIMT [7], simple measurements of gain were added to confirm effectiveness of the intervention. Range of motion was tested for shoulder abduction and external rotation, elbow flexion, extension, and supination, and wrist extension with the child in a fully supported supine or sitting position using a goniometer. Strength was measured for shoulder abduction and external rotation, elbow flexion and extension, and wrist extension using a dynamometer. The best of three supported attempts served as the outcome variable. These measures are reported in Table 2.

### 2.4. EEG Acquisition and Preprocessing

A paradigm allowing differentiation of brain responses to a calibrated light touch stimulus consisted of a puff of air to the pad of the distal phalanx of the index finger. To verify that the observed ERP effects are specific to the sense of touch, ERPs were also recorded to a “sham” stimulus, with delivery of the identical puff but with the nozzle turned away from the hand (as previously published, [5]). A custom apparatus delivered 60 puff and 60 sham stimuli randomly, with varying intertrial intervals of 2000–2500 ms, with no more than 2 consecutive puff stimuli to prevent habituation. Randomization was applied in terms of puff vs. sham stimulation, with one hand tested at a time due to the apparatus abilities to deliver one puff and one sham. The order of hand stimulation was counterbalanced. Stimulus delivery was controlled by E-Prime (version 2.0; Psychology Software Tools Inc., Pittsburgh, PA, USA).

Continuous EEG was recorded with a 128-channel Geodesic Sensor Net connected to a NetStation amplifier (software version 4.3; Electrical Geodesics Inc., Eugene, OR, USA). Data were sampled at 1000 Hz with online filters set to 0.1 Hz high-pass and 400 Hz low-pass. Electrode impedances were kept below 50kΩ and were checked at the start of each block of trials. All electrodes were referenced online to Cz and rereferenced offline to an average reference. The continuous data were filtered offline using a 0.3 Hz high-pass and 40 Hz low-pass filters and then segmented on stimulus onset to span a 200 ms prestimulus baseline and a 700 ms poststimulus interval. Resulting segments were screened for motor/ocular artefacts using standard algorithms included in NetStation, followed by a manual review based on visual inspection. Data for electrodes with poor signal quality were interpolated using 3D splines, with an average of 12 channels interpolated (range 5–16 channels). Interpolation was conducted for each child's data considering all conditions and measurement points collectively so that the same channels were interpolated for all conditions.

To obtain ERPs, the artefact-free epochs were averaged and prestimulus baseline corrected for all subjects and each of the eight conditions including type of stimulation (touch vs. sham), stimulated hand (more vs. less affected side), and the ERP recording session (pre- vs. postintervention). For a child's dataset to be included in the statistical analyses, all individual conditions had to include ≥12 usable trials. For touch stimuli, the average (±SD) number of trials included per session and hand was 21 ± 8 (more affected, preintervention), 20 ± 7 (less affected, preintervention), 20 ± 9 (more affected, postintervention), and 23 ± 9 (less affected, postintervention). These values did not reliably differ (*F* = 1.4; *p* > 0.26). For sham stimuli, the average (±SD) number of trials included per session and hand was 19 ± 7 (more affected, preintervention), 21 ± 8 (less affected, preintervention), 21 ± 8 (more affected, postintervention), and 20 ± 7 (less affected, postintervention). These values did not reliably differ (*F* < 1).

To account for ERP differences associated with the left vs. right more affected side, data from all participants were relabeled to fit them to a common laterality map, where brain activity contralateral vs. ipsilateral to the more affected hand was anchored to the same hemisphere across all participants, independently of the side of their impairment (see [12] for a similar procedure).

### 2.5. Analysis of ERPs

**Figure 1:**
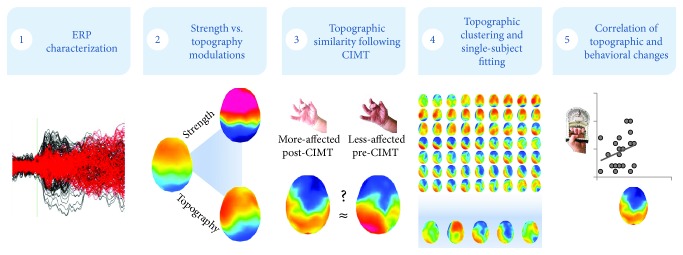
Flowchart of analytical steps performed in the present study. A multistep analysis framework was developed to identify the mechanisms within the somatosensory brain system contributing to impairments as well as (post-CIMT) to the recovery of motor function. In step 1, we compared ERPs to puff (and sham) stimuli to the more and less affected hand before and following CIMT. In step 2, we investigated whether CIMT modulated the strength of responses within statistically indistinguishable brain networks and/or through alternations in ERP topography. In step 3, we performed spatial correlations on ERPs to investigate whether CIMT influenced ERP topography by forcing the somatosensory system controlling the more affected hand to activate patterns of brain activity (“template maps”) more similar to those controlling the less affected hand. In step 4, to understand whether the observed associations were driven by relatively longer involvement of the more functional patterns of somatosensory brain activity, we, first, submitted group-averaged ERPs to a hierarchical cluster analysis and then fit the template maps detected in group-averaged ERPs to single-subject ERPs to test whether they were reliably present. Lastly, in step 5, we performed correlations between relative duration of the more functional template maps and relative changes in scores on the sensory and motor diagnostic CP tests, to test behavioral associations with observed patterns of somatosensory activity.

An overview of our multistep analysis is detailed in Figure 1.

#### 2.5.1. Characterization of Somatosensory ERPs in Children with CP (Step 1)

To bridge the present electrical neuroimaging analyses with results in prior studies, we visualized the data from topographic maps in 100 ms intervals over the poststimulus period. Given the reference-dependent nature of analyses of voltage waveforms, we restricted our subsequent quantitative analyses to reference-independent measures of ERP.

#### 2.5.2. Specification of Neurophysiologic Changes after CIMT on Somatosensory ERPs (Step 2)

Electrical neuroimaging analyses were performed as previously described [10]. First, we assessed whether CIMT influences ERPs in the more affected hand by altering the strength of response to calibrated touch within a statistically indistinguishable configuration of sources. As before, we were equally interested whether similar alterations would be observed as a function of CIMT in the less affected hand. Response strength in ERPs across the two hands before and after CIMT was quantified using reference-free Global Field Power measures (GFP), calculated as the root mean square, or standard deviation, across the electrode montage. GFP was analyzed using a 2-way Session × Side ANOVA for each peri-stimulus time sample using STEN tools (STEN 1.0 Statistical Toolbox for Electrical Neuroimaging, http://doi.org/10.5281/zenodo.1164038). Then, we assessed whether CIMT influenced ERP topography for the more and less affected hand. These ERP topography differences were quantified using reference-free Global Dissimilarity (DISS), the root mean square of the differences between two GFP-normalized vectors (here the 128-channel ERPs from any given pair of conditions). DISS values range from 0 to 2, with 0 indicating no topographic differences and 2 indicating topographic inversion. DISS was analyzed in a factorial design using the Randomization Graphical User interface (RAGU; [23]) to perform nonparametric tests comparing the observed value to an empirical distribution based on permutations of the data from all participants/conditions. The 2-way Session × Side ANOVA conducted on the DISS values revealed time periods of statistically significant changes in topography in the analysis involving puff, but not the sham, stimuli (see Results). Thus, and as detailed below (Sections 2.5.3–2.5.5), CIMT acted exclusively on the topography of ERPs to calibrated puff stimuli, indicating differences in configuration of the underlying neural generators [10]. Therefore, all next steps focused on identifying the nature of these spatial modulations.

Changes in ERP topography are forcibly the result of changes in the underlying configuration of active sources. Therefore, this global analysis of the ERP topography allowed for a direct neurophysiologic interpretation as to the root cause of differences across either hands or pre- vs. postintervention. It also allowed statistically based inferences regarding whether CIMT resulted in a change in ERP response strength and/or a change in the brain network generating the ERP response to tactile stimuli.

#### 2.5.3. Verifying Similarities between More and Less Functional ERP Topography (Step 3)

Spatial correlations were computed between the puff response from the pre-CIMT intervention less affected hand condition and (1) the post-CIMT intervention more affected hand in order to assess potentially positive effects of CIMT (i.e., “more functional”) and (2) the post-CIMT intervention in the same, less affected hand, in order to assess potentially negative effects of CIMT (i.e., “less functional”). Spatial correlation is inversely related to DISS (see e.g., [10] for the formula) and was calculated solely for puff stimuli in the moment-by-moment scalp topography across these pairs of ERPs. The results of this analysis indicated the poststimulus timing of more functional and less functional effects.

#### 2.5.4. Identifying the Nature of More Functional Patterns of ERP Topography following CIMT (Step 4)

Given that the preceding steps identified the presence of topographic differences between conditions, it was then important to determine whether these differences stemmed from alternations in the sequence of occurrence of the same set of template maps or, alternatively, from template maps that were uniquely characteristic of some conditions. To do this, we first submitted the group-averaged ERPs across the four Session × Side conditions to a topographic analysis based on a hierarchical clustering algorithm [10]. This analysis identifies the temporal pattern of stable electric field topographies (“template maps”) both within and between conditions, which can then be contrasted by their mean duration. The clustering is exclusively sensitive to topographic modulations because data are first normalized by their instantaneous GFP. The optimal number of temporally stable ERP clusters (i.e., the minimal number of maps that accounts for the greatest variance of the dataset) was determined using a modified Krzanowski-Lai criterion [10]. The clustering made no assumption regarding the orthogonality of the derived template maps. Clustering analysis was conducted for the four puff stimulus conditions within the 250 to 550 ms time window because this window was the only one with a statistically significant spatial correlation between the preintervention unaffected side and the postintervention affected side ERPs. The implication is that this time period represents increased functional processing of calibrated touch post-CIMT. Three template maps were identified in the group-averaged ERPs across the full 2 × 2 design within the 250–550 ms time window. To assess whether they were reliably present in the single-subject ERPs, these three maps were then submitted to a fitting procedure, where each time point of each single-subject ERP is labelled according to the template map with the best spatial correlation. To statistically assess whether CIMT increased the relative duration of more functional patterns of activity in the moment-by-moment scalp topography of the ERP in the more affected hand and reduced it in the less affected hand, a 3-way ANOVA with within-subject factors of Map, Session, and Side was then carried out. To provide a direct test of whether the intervention induced the stronger involvement of more functional patterns of somatosensory activity, the fitting analyses focused on comparing duration of the maps present in the ERP from the preintervention less affected hand condition to their duration in the ERP at the more affected hand postintervention. At the same time, pre- and post-CIMT differences in the relative duration of the template maps were assessed also within the less affected hand in order to test for the effects of CIMT as reducing functional patterns of activity. We focused only on those template maps that were clinically relevant in that they reliably differed in their duration pretreatment between the less and more affected hand.

#### 2.5.5. Associations between ERP Topographic Measures and Sensory Assessments (Step 5)

Pair-wise single-tailed *t*-test (based on published data) compared differences between sides for all sensory measures. To assess whether the relative increases in duration of the more functional pattern of somatosensory activity were behaviorally relevant, we performed nonparametric correlations (Spearman's rho) between those behavioral measures showing significant differences across the two hands pre-CIMT and the relative duration of the clinically relevant ERP template map pre- versus post-CIMT. We first confirmed the independence of values between the hands before entering data from the two hands into a single correlation analysis. There was no reason to assume that ERP measures would correlate differently with a more or a less affected hand. The measures from the two hands were taken as individual data points and analyzed in a pair-wise manner with their corresponding ERP values. In this way, we avoided spurious correlations from assuming that ERP could only correlate with behavioral function measurements in a less affected extremity. The same results were observed with these analyses as well as with repeated-measures correlation analyses specifically developed to accommodate correlation analyses on data with interrelated within- and between-subject variance [24]. All significance thresholds were set to alpha = 0.05.

## 3. Results

### 3.1. Somatosensory ERPs in Children with CP (Step 1)

**Figure 2:**
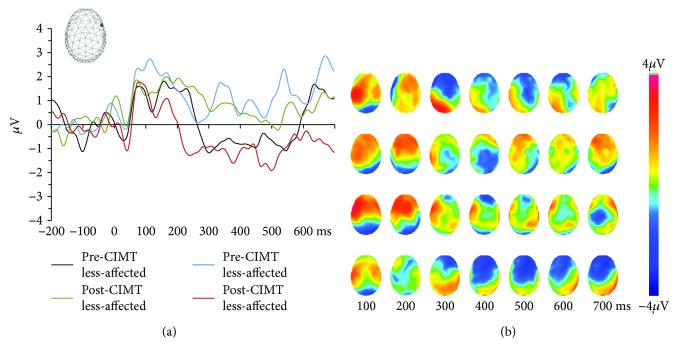
Group-averaged ERPs from an exemplary electrode and accompanying sequential topographic maps over ~250–550 ms poststimulus. (a) Group-averaged ERPs from a right fronto-central scalp site (see inset) in response to all Side and Session conditions. Note the similarity of responses to the pre-CIMT less affected and post-CIMT more affected conditions as well as the responses to the pre-CIMT more affected and post-CIMT less affected conditions, particularly over the ~250–550 ms poststimulus period. (b) Sequential topographic maps (top view with left hemiscalp on the left and frontal regions upwards) of ERPs from all Side and Session conditions shown at 100 ms intervals over the poststimulus period. Again, note the same pattern of similarity as in (a).

Figure 2 displays ERPs from a representative lateral fronto-central scalp site as well as the sequence of topographic maps at 100 ms intervals for each Side and Session condition. Responses to stimulation of the more versus less affected hands over the ~200–300 ms poststimulus time window prior to CIMT appeared to differ, consistent with previous findings of altered somatosensory processing in CP [5]. Responses to the more affected hand post-CIMT resembled those of the less affected hand pre-CIMT, while responses to the less affected hand post-CIMT resembled those of the more affected hand pre-CIMT. This pattern was particularly apparent over the ~250–550 ms poststimulus interval.

### 3.2. Specifying Neurophysiologic Mechanisms of Effects of CIMT on Somatosensory ERPs (Step 2)

There was no evidence that the effects of CIMT on ERPs across the two hands were driven by alterations in the overall strength of somatosensory response patterns. The 2-way Session × Side ANOVA on the GFP time series revealed no significant effects at any latency in the −100 to 700 ms peri-stimulus time period. In contrast, a randomization-based analysis of the ERP topography (using DISS) revealed multiple time windows where a two-way Session × Side interaction lasted for minimally 20 ms [25]: 205–234 ms, 289–312 ms, 321–360 ms, and 482–510 ms poststimulus. Thus, intervention effects on ERPs appeared driven by alterations in the topography of electric fields during somatosensory responses.

### 3.3. Verifying Similarities between More and Less Functional ERP Topography (Step 3)

**Figure 3:**
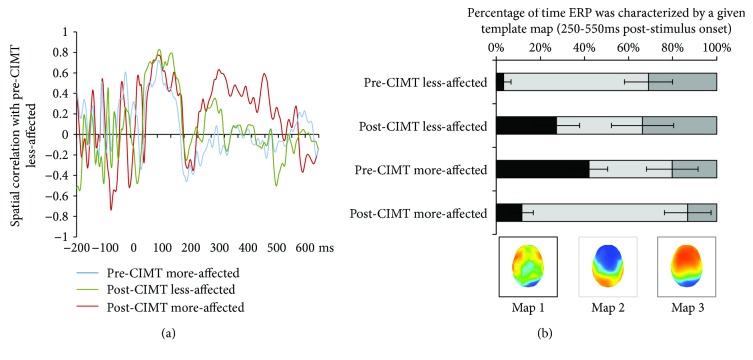
CIMT-induced effects on ERP topography across less and more affected hand. (a) Spatial correlation between ERPs from the pre-CIMT less affected hand and each of the other Side and Session conditions as a function of time. Note that while there is high positive spatial correlation over the initial ~200 ms poststimulus onset across all conditions, only the post-CIMT more affected hand ERP exhibited a high spatial correlation with the pre-CIMT less affected hand ERP over the ~250–550 ms poststimulus period. Both other conditions exhibited spatial correlations near 0. (b) Hierarchical topographic cluster analysis identified 3 template maps that characterized the 250–550 ms poststimulus period across all conditions (see insets). Single-subject fitting based on spatial correlation between these template maps and individual ERPs yielded the average percentage of time each of these three template maps characterized each condition. The bar graphs show that Map 1 was both clinically relevant and also that its presence changed in a manner consistent with beneficial plasticity; its mean duration decreased in the more affected hand post- vs. pre-CIMT. The presence of Map 1 also demonstrated a nonsignificant trend for maladaptive plasticity; its mean duration increased in the less affected hand post- vs. pre-CIMT. The graphs concerning Map 2 show that despite differing pre- vs. post-CIMT patterns for the less affected and more affected hands, there was no reliable evidence that the presence of this map was clinically relevant. The presence of Map 3 did not differ across conditions.

Because DISS is a measure of topographic difference rather than similarity, analyses focused on spatial correlation between the reference ERP topography, i.e., the less affected hand pre-CIMT vs. each of the other conditions. All conditions exhibited robust correlations with the reference ERP topography over the ~50–200 ms poststimulus time window. By contrast, in a subsequent time period (~250–550 ms), only the ERP topography in response to stimulation of the more affected hand post-CIMT correlated with the reference condition, with spatial correlations involving the other conditions remaining near 0 (Figure 3(a)). These results suggest that ERPs to touch at the more affected hand post-CIMT intervention exhibited topographic distributions more similar to those in the ERPs of the less affected hand pre-CIMT intervention.

### 3.4. Identifying the Nature of More Functional Patterns of ERP Topography following CIMT (Step 4)

The topographic cluster analysis was guided by the time periods where increasing similarities in ERP topography between reference ERP and that in the more affected hand post-CIMT. Over the 250–550 ms poststimulus time period, there were three distinct patterns of activity, differently present in the four ERP conditions (Figure 3(b)). The fitting procedure showed a significant 3-way interaction (*F*_(2, 8)_ = 7.14; *p* = 0.017; *η*_p_^2^ = 0.64). In light of this interaction and our specific research questions, we focused on identifying those template maps that were clinically relevant (see analysis section). This was achieved by submitting the fitting results for each map to a 2 × 2 ANOVA.

For map 1, there was a significant interaction between Session and Side (*F*_(1, 9)_ = 16.05; *p* = 0.003; *η*_p_^2^ = 0.64). The presence of this topographic pattern was clinically relevant; it differentially characterized the pre-CIMT responses to the more affected and less affected hands (*t*_(9)_ = 3.76; *p* = 0.004) (Figure 3(b)). The mean duration of this map's presence decreased in the more affected hand following CIMT (*t*_(9)_ = 2.48; *p* = 0.04). Conversely, the mean duration of map 1 increased in the less affected hand following CIMT (*t*_(9)_ = 2.22; *p* = 0.054). For map 2, there was a significant interaction between Session and Side (*F*_(1, 9)_ = 8.43; *p* = 0.017; *η*_p_^2^ = 0.484). However, there was no evidence that the relative presence of this map was clinically relevant (*p* > 0.10). For map 3, there was no evidence of significant interaction between Session and Side (*F*_(1, 9)_ < 1). Therefore, in Step 5, we focused exclusively on map 1 given this evidence of clinical relevance as well as plasticity in a potentially positive direction for the more affected hand.

### 3.5. Associations between ERP Topographic Measures and Neurosensory Functional Assessments (Step 5)

Mirroring our ERP analyses, we first identified those sensory measures that reliably differentiated between the less and more affected hands before CIMT. Therein, stereognosis, grip, and pinch tests all revealed reliable differences between more affected and less affected hands before CIMT, *p*'s < 0.011 (see Table 3). Similarly, stereognosis, grip, and pinch tests all revealed reliable differences across the more affected and less affected hand, when tested *post*treatment (see Table 2). As detailed in previous experiments, map 1 reflected a less functional pattern of neural activity. Based on those results, the ERP-behavior relationships were assessed between the amount of time the pre-CIMT ERP was characterized by this less functional topographic pattern and scores on each of these measures of sensory-motor function, regardless of the hand.

A negative relationship was observed for all three neurosensory measures, but it was statistically significant only for grip and pinch tests (rho_(18)_ = −0.634, *p* = 0.003 and rho_(18)_ = −0.581, *p* = 0.007, respectively) and not for stereognosis (rho_(18)_ = −0.404, *p* = 0.077). In a final step, we tested whether CIMT-induced changes in either of the two neurosensory measures were associated with changes in time spent in the clinically relevant ERP pattern. Decreased time spent in map 1 during tactile processing post-CIMT was correlated with an improved grip across both hands (rho_(18)_ = 0.648; *p* = 0.002). Among all of the sensory and sensory-motor measures, only grip strength in the more affected hand was modulated by CIMT (*t*_(9)_ = −6.30; *p* < 0.001). No other measure significantly changed (all *p* > 0.1).

### 3.6. Control ERP Analyses: Sham Stimuli

Analyses of ERPs triggered by sham, i.e., nonsomatosensory, stimuli provided no indication of reliable differences between the more affected and less affected hands nor any effect of CIMT either in terms of ERP strength or topography.

## 4. Discussion

The current study provides novel insights into brain mechanisms of hard-constraint CIMT effects on sensory and sensory-motor function in young children with hemiplegic CP. In the more affected hand, CIMT may reduce the recruitment of less functional brain networks during stages of somatosensory processing over the 250–550 ms time period. The magnitude of CIMT-induced behaviorally measured sensory improvements correlated with the attenuation of relatively less functional somatosensory activity patterns. Lastly, CIMT appears to also alter sensory processing in the less affected hand, where it may also increase the relative involvement of less functional patterns of brain activity in response to somatosensory stimulation.

Impairments in somatosensory function alter the ability of children with CP to obtain information for exploration of their environment, diminish awareness of their body in space, and contribute to alterations in sensory-motor functions such as grip forces and reach precision [18, 26–28]. Functionally, attenuated oscillatory activity has been consistently reported as distinguishing the more versus less affected somatosensory cortices in hemiplegic CP [3, 9]. These impairments predicted motor performance across a wide range of functions, supporting the concept of increased noise within the somatosensory system as one of the neurophysiologic mechanisms linking somatosensory and motor deficits [1, 3, 29]. Complementary research demonstrated that somatosensory ERPs exhibit clinical sensitivity, revealing differences between more and less affected extremities in hemiplegic CP. ERPs to calibrated touch could distinguish between hands where the motor function was less versus more impaired [5]. In the current study, we extended these findings by investigating whether somatosensory processing in CP can measure improvements in sensory-motor functions in the more affected extremity following an evidence-based intervention. Simultaneously, we tested whether electrical neuroimaging analyses [10, 11] could identify further neural mechanisms within the somatosensory system that underlie the recovery of sensory-motor functions in the more affected extremity.

Touch-induced ERPs differentiated between more and less functional hands, pre- vs. post-CIMT. This was not the case for ERPs to sham stimuli, suggesting that the effects of the intervention are specific to the somatosensory system. In the more affected hand, a short intensive CIMT course altered the topographic pattern of somatosensory responses, so that it more closely resembled (i.e., correlated with) the pattern observed in the less affected hand before treatment. Changes in the topography of neural activity visible on the scalp necessarily flow from changes in the configuration of activated brain areas [10, 11]. Thus, our findings suggest that CIMT may significantly help overcome tactile dysfunction of the more affected extremity by facilitating activation of brain networks similar to those activated by the hemisphere controlling the less affected extremity. One of many possible mechanisms for observed CIMT effects could then be improvement of sensory-motor functions, by a relative decrease in prevalence of less functional patterns of brain activity in the less functional extremity.

### 4.1. Somatosensory Changes after CIMT in the Less Affected Extremity

A secondary theoretical aim of our study was to test whether CIMT may also alter more functional patterns of somatosensory brain activity in CP in a less affected limb. Because constraint-based methods are relatively low-cost/low-technology interventions with demonstrated efficacy, they remain attractive potential rehabilitation tools. At the same time, the constraint use may have unintended profound consequences on sensory processing and even motor function of a previously less impaired extremity, especially in the developing brain. There is mounting evidence that the function of both upper limbs is adversely affected in adult hemiplegia, even in the presence of a clearly unilateral brain lesion (e.g., [30]). Consideration of the less affected limb during CIMT is even more pertinent in infancy and childhood, where sensory afferents and associative motor pathways and functions are still developing, and plasticity is high [31]. Furthermore, the less affected limb in hemiplegic children has significantly worse performance compared to the nondominant (i.e., worst) limb of typically developing children on a number of measures. It is therefore critical not to worsen its function, even inadvertently. Thus, sensory-motor brain systems should be shielded from potentially negative influences as much as possible (see [15] for impact of painful experiences on early somatosensory processing).

We observed that the reduction in motor and sensory inputs after prolonged and continuous constraint was associated with alterations in somatosensory processing of the less affected extremity towards a less functional ERP pattern. Following CIMT, brain processes governing activity at latencies beyond 200 ms poststimulus within the less affected hemisphere demonstrated patterns of ERP activity that were more prevalent (present for a longer period of time) upon stimulation of the more affected hand at baseline. Nevertheless, these findings should be qualified. For one, these changes did not always correlate reliably with behavioral assessments of sensory functions in our cohort of young children. Rather, they correlated with assessments involving sensory-motor function. Additionally, quantifiable differences in somatosensory processing in the less affected hand could prove temporary, as no adverse sensory symptoms were identified using behavioral measures or according to parent estimation 6 months later. Similarly, no adverse effects of CIMT on motor functioning of the less affected hand were reported in a study of adult stroke patients, though adverse effects on cortical representation of the muscle were reliable albeit transient [32] While it would be necessary to replicate these changes in somatosensory processing in a large long-term prospective randomized controlled trial, our findings provide the first preliminary evidence of potentially unintended impact of CIMT on somatosensory brain function. Thus, the constraint parameters (timing, cast rigidity/removability) as well as the structure of rehabilitation (constraint alone/with bimanual training) in childhood hemiplegia may need to be carefully evaluated for possible effects on the less affected hand [17]. In the meantime, modifications may be considered in current clinical practice, such as restraint forms that do not deprive the less affected extremity of sensory feedback and gross movements [33]. Timing of hard-constraint rehabilitation onset may need to be limited to time periods well past a critical window of somatosensory development in infancy, while restraint duration may need to be shortened to prevent the development of potential asymmetric and unbalanced somatosensory associations. In turn, combining constraint and bimanual therapy [34] may prove most effective in restoring cross-hemispheric connections and facilitating integration of information across the sensory-motor nervous systems (echoing the most promising rehabilitation approaches in amblyopia; e.g., [35]). A further important consideration is that use of the more affected hand will likely need to be encouraged after removal of the cast on the less affected hand to minimize long-term overdependence on the less affected hand postintervention [36]. We are currently investigating whether the present findings are replicated in a larger sample of infants 6–24 months of age, using a modified CIMT approach to ensure safety of patients with limited attentional capacity and at a time of heightened neural plasticity. If successful, these findings would support the feasibility of ERPs and electrical neuroimaging as a valuable wide-ranging assessment tool in CP diagnostics and rehabilitation assessment. Moreover, these results may have important implications for treatment of children with developmental disregard where links between sensory and attention-related processes pertaining to motor control seem to be compromised [37, 38].

### 4.2. Measurement of Somatosensory Changes Using Behavioral Measures in Young Children

Abnormal processing of somatosensory stimuli is prevalent in children with CP [2, 39] and drives poor cortical feedback during performance, resulting in imprecise learning of movement that in turn leads to imprecise or incorrect inferential data during future movements. In the present study, topographic changes in ERPs to calibrated touch after CIMT were related to changes in scores on tests measuring sensory-motor functions, as assessed across both hands. Only grip demonstrated this significant correlation while pinch showed a similar, albeit nonsignificant, correlation. Stereognosis, while reliably differentiating more and less affected hand pre-CIMT, did not correlate with changes in the less functional topographic ERP patterns when measured as a pre- vs. post-CIMT difference. Many behavioral measures of sensory function, such as single-point localization or stereognosis, were developed in older children and require cognitive processing due to their task-based nature. Conversely, pinching or grasping an object until perceived resistance neither requires verbalization nor involves deliberate recruitment of attentional networks. Such measures as stereognosis and/or grating orientation tasks were recently shown to improve with both intensive CIMT and bimanual training [36, 40]. However, those studies were carried out in children older (>8 years of age) than those tested here, and even therein, stereognosis improvements were not always reliable. This underlines the potential viability of the recording of somatosensory ERPs in combination with electrical neuroimaging analyses as an important assessment tool in cerebral palsy, especially in younger populations. In addition to a possible effect of young age (and associated variability) in our cohort, the duration of CIMT may have been too short to affect behavioral measures of function. This has been hypothesized in trials involving other rehabilitative strategies in which 4 weeks of enhanced sensory exposure did not result in increases of sensory measures above baseline changes related to hand use [20]. Our present results show that ERPs may detect CIMT-induced changes prior to their consistent appearance in behavioral measures, particularly when obtained from young children.

### 4.3. Electrophysiology in the Study of Mechanisms & Rehabilitation in Pediatric CP

Besides providing novel insights into possible mechanisms of plasticity in CP, our findings point to ERPs as an efficient technique to quantify treatment effects in CP. Somatosensory cortical responses can be measured using high-density EEG recordings and electrical neuroimaging in children with CP and do not require active participation, joint, or sustained attention. Neuroimaging can reveal treatment-relevant effects in brain structure and function [41], including in CP [42]. Traditional sensory and behavioral tests in stroke patients demonstrate that rehabilitation can lead to compensation using residual capacities (by the less affected limb or alternative muscle groups of the more affected side) [43] rather than to the recovery of lost/suppressed functions. Interpretation of fMRI results for determining reorganization or neurological restoration vs. compensation, the use of preexisting redundancies, and development of new strategies can all be problematic (e.g., [44]). Thus, methods recording direct physiological activity of the brain, such as MEG and EEG, combined with advanced signal processing techniques could prove especially useful. Electrical neuroimaging has previously revealed temporal and spatial mechanisms governing brain plasticity in health (e.g., [12–14]) and in disease (e.g., cross-modal reorganization following specific language impairment, language in CP; [16, 45]).

In our study, the electrical neuroimaging approach has provided several novel clinically relevant insights in the area of CP: (1) It replicated the clinical asymmetry of somatosensory ERPs across the less and more affected extremities (cf. [5]); (2) It demonstrated for the first time plasticity of somatosensory brain processes within the more affected somatosensory system following CIMT; (3) It revealed effects of CIMT on the somatosensory-motor functions in the less affected hand; and (4) It provided evidence for the mechanism of action for CIMT: activating somatosensory brain networks within the more affected hand (after CIMT) similar to those activated within the less affected hand (as measured at baseline).

It is now well established that cerebral palsy is characterized by substantial impairments in somatosensory processing in the more versus less affected proximity that have clear links with behavioral deficits. However, the techniques employed by some of the extant studies may not be best suited to pediatric and moreover clinical pediatric populations (e.g., MRI or MEG that requires participants to remain immobile and is costly to use). We demonstrate here is the feasibility of high-density EEG and electrical neuroimaging analyses as an assessment tool for somatosensory function in cerebral palsy, especially in younger children, that capitalizes on the cost effectiveness and ease-of-use of EEG in combination with state-of-the-art, yet intuitive and mathematically simple, signal processing analyses. The validity of our results is supported by several lines of evidence. First, the latency of the observed modulations of the somatosensory evoked potentials (SEPs) is highly consistent with the literature; SEPs extend over time to at least 300–400 ms poststimulus onset even when recorded intracranially from subdural electrodes on the central sulcus (e.g., [46]; see also [47]). We would note that the effects we report here occur at similar latencies as in a study of neonates following electrical median nerve stimulation [47]. Moreover, our latencies are similar to those observed in studies of children involving a highly similar experimental setup to that used here (e.g., [5], which was a study of children with cerebral palsy, as well as in [48], which was a study of children with ASD). The data suggested that CIMT impacted responses to the more and less affected hands in opposing manners. Second, SEPs were time-locked and phase-locked to the onset of the air puff. Thus, there are strong bases to conclude that the observed CIMT-induced effects on SEPs are indeed related to somatosensory processes. Third and relatedly, our novel mechanistic insights are independent of claims regarding their specific locus (e.g., limited to somatosensory cortices). If one wanted to make such claims, this would require source estimation analyses, which would in turn necessitate obtaining high-resolution anatomical MRIs, to model the variable location of lesion(s) in our participants. In our study, as frequently in clinical practice, obtaining such images was not feasible. Notwithstanding, the heterogeneity in the underlying etiology of the cerebral palsy would likely render source estimations intractable because the lead field matrices would not be uniform (at least at a group level; see also Table 1). Nevertheless, we provided several novel insights into the mechanisms whereby CIMT might improve touch processing, which demonstrates the utility of approaches, such as ours, focusing on the surface EEG (i.e., dense EEG montages) data alone, in clinical settings.

Relatedly, electrical neuroimaging, in contrast to the majority of clinical EEG investigations, employs measures that are not dependent on the employed reference. This makes our results robust, in that the same result would be obtained irrespective of the reference used by a given laboratory or researcher [10]. In turn, this robustness enables findings from different teams to be directly compared. This is not possible currently, as reference-dependent measurements dominate EEG research, especially in the clinical domain.

The current study has several limitations. First, the sample was relatively heterogeneous. Because effects were noted despite this limitation, functional patterns of activity may be more clinically relevant than the nature or the location of the lesion in the brain. The sample size was small but comparable to those in the previously published MEG work [9, 39] or other studies on CP deficits or, notably, treatments [49]. The sensitivity of topographic patterns of somatosensory activity persisted between hands and sessions in this small group. The findings in the less affected hand could be taken as discordant with some models (e.g., [50]) assuming that the early perinatal nature of the brain insult would “push” the brain of CP children onto developmental trajectories different from those in the typically developing population. However, a comparison with a typically developing group in this instance would not be as strong as employing each child participant as their own control: the presence of focal, large, and distorting lesions within the brains of children with CP would make comparisons with similar spatio-temporally covarying brain networks unrealistic.

The potential mechanism of CIMT (at least for the more affected hand) may entail the unmasking of preexisting but suppressed “healthy” connections. This general idea is well corroborated by the explanations put forward for ascending rehabilitation approaches to other lateralized neurological disorders, such as prism adaptation in stroke or dichoptic games in amblyopia [35]. Here, the high intensity of the rehabilitation regime might be key in providing the necessarily strong and consistent signal for functional reorganization within the impaired somatosensory system, e.g., by increasing synaptic efficiency via Hebbian learning mechanisms [51]. Consistent with this, >60-hour-long intervention spanning a week has been revealed to be especially effective in motor function recovery in a meta-analysis of multiple studies spanning >100 children [18]. The combination of bimanual tasks with the constraint of the less affected extremity might provide the necessary balance of activity across both the lower-level sensory (controlling the early processing in the affected proximity) and the intact domain-general systems for cognitive control, whose coordination might be crucial for optimal recovery from this and other lateralized neurological disorders [52]. The feedback activity within relevant sensory systems might be as important as bottom-up activity within the early sensory system, in addition to top-down, feedback input from domain-general brain systems; a pattern consistent with the timing of the present results. Cognitive control systems might respond in a phasic, transient, and task-dependent manner to the increased “effort” when damaged downstream domain-specific sensory networks convey signals, but they are likely responsible at the same time for having tonically downregulated processing of signals emerging from the impaired networks before the treatment [53].

## 5. Conclusion

To summarize, while in need of replication with a larger sample size, our present results offer novel mechanistic insights into the etiology and plasticity in hemiplegic CP. We replicated and extended the clinical sensitivity of somatosensory ERPs (i.e., ability for touch-induced ERPs to distinguish between more affected and less affected hands), suggested unintended effects of CIMT on the less affected extremity, and quantified the likely neurophysiological mechanisms governing brain/sensory feedback plasticity. While they demonstrate the effects of CIMT, our results importantly support the viability of dense EEG and electrical neuroimaging as a novel in CP assessment tool for somatosensory function, especially for younger populations.

## Figures and Tables

**Table 1:** Characteristics of cerebral palsy in the tested patients.

Subject	Sex	Age	More affected side	MACS	Preterm (<37 weeks)	Findings on clinical neuroimaging
1	M	8	R	2	0	Partial agenesis of the left cerebellar hemisphere with mild rotational shift of the brainstem
2	F	6	L	3	0	Prominent neuronal migration disorder in the right hemisphere with dysgenesis
3	F	8	L	1	1	Right temporal cystic encephalomalacia
4	M	6	L	2	1	Left frontal parietal IVH, grade IV
5	M	6	L	2	0	Bilateral IVH grade III and prematurity with ventricular dilation, right PLIC
6	M	8	L	2	1	Right IVH grade IV, right PLIC
7	F	6	R	1	0	Stroke, left PLIC
8	F	5	L	1	1	Bacterial meningitis 36 weeks MRI, diffuse injury more marked on the right hemisphere
9	F	5	R	2	0	Ischemic lesions, left more prominent than right basal ganglia affected, PLIC not affected
10	M	7	R	1	0	Left subarachnoid and subdural hemorrhages

PLIC: posterior limb of the internal capsule; IVH: intraventricular hemorrhage; MACS: manual abilities classification scores.

**Table 2:** Results of the neurosensory tests as carried out for the more and less affected hand before the CIMT intervention.

Test	Hand	Unit	Mean	Std. deviation	*p* value
Somatosensory registration	More affected	Semmes monofilament force in grams	3.13	.524	.524
	Less affected		2.99	.329	

Single-point localization	More affected	# correctly identified localisations (max. 4)	2.50	.707	0.264
	Less affected		2.80	.422	

Two-point discrimination	More affected	Distance in millimeters	4.90	2.079	0.046
	Less affected		3.00	1.886	

Stereognosis	More affected	# correctly identified pairs (max. 9)	5.10	2.644	0.011
	Less affected		7.70	1.160	

Pinch	More affected	psi	1.800	.9189	0.001
	Less affected		3.400	.7746	

Grip	More affected	psi	4.1	2.1187	<.001
	Less affected		9.25	3.2081	

**Table 3:** Results of the neurosensory tests as carried out for the more and less affected hand after the CIMT intervention.

Test	Hand	Unit	Mean	Std. deviation	*p* value
Somatosensory registration	More affected	Semmes monofilament force in grams	2.83	.01	.343
	Less affected		2.83	.00	

Single-point localization	More affected	# correctly identified localisations (max. 4)	2.90	.32	—
	Less affected		2.90	.32	

Two-point discrimination	More affected	Distance in millimeters	3.89	1.90	.037
	Less affected		2.22	.67	

Stereognosis	More affected	# correctly identified pairs (max. 9)	5.30	2.79	<.025
	Less affected		8.20	1.14	

Pinch	More affected	psi	1.88	.78	<.001
	Less affected		3.30	1.06	

Grip	More affected	psi	4.1	2.28	<.007
	Less affected		7.10	2.81	

## Data Availability

The data used to support the findings of this study are available from the corresponding author upon request.
